# Bioactive Copper-Doped Glass Scaffolds Can Stimulate Endothelial Cells in Co-Culture in Combination with Mesenchymal Stem Cells

**DOI:** 10.1371/journal.pone.0113319

**Published:** 2014-12-03

**Authors:** Subha N. Rath, Andreas Brandl, Daniel Hiller, Alexander Hoppe, Uwe Gbureck, Raymund E. Horch, Aldo R. Boccaccini, Ulrich Kneser

**Affiliations:** 1 Department of Plastic and Hand Surgery, University of Erlangen-Nürnberg, Erlangen, Germany; 2 Department of Biomedical Engineering, Indian Institute of Technology Hyderabad, Yeddumailaram, Telangana, India; 3 Institute of Biomaterials, Department of Materials Science and Engineering, University of Erlangen- Nürnberg, Erlangen, Germany; 4 Department for Functional Materials in Medicine and Dentistry, Universtiy of Würzburg, Würzburg, Germany; 5 Department of Hand, Plastic and Reconstructive Surgery - Burn Center, University of Heidelberg, Ludwigshafen, Germany; 1 Biomaterials for Regenerative Therapies Group, Institute for Bioengineering of Catalonia, Baldiri Reixac 15–21, Barcelona 08028, Spain, 2 Technical University of Catalonia, Av. Diagonal 647, Barcelona 08028, Spain, 3CIBER-BBN, María de Luna 11, Zaragoza 50, Spain

## Abstract

Bioactive glass (BG) scaffolds are being investigated for bone tissue engineering applications because of their osteoconductive and angiogenic nature. However, to increase the *in vivo* performance of the scaffold, including enhancing the angiogenetic growth into the scaffolds, some researchers use different modifications of the scaffold including addition of inorganic ionic components to the basic BG composition. In this study, we investigated the *in vitro* biocompatibility and bioactivity of Cu^2+^-doped BG derived scaffolds in either BMSC (bone-marrow derived mesenchymal stem cells)-only culture or co-culture of BMSC and human dermal microvascular endothelial cells (HDMEC). In BMSC-only culture, cells were seeded either directly on the scaffolds (3D or direct culture) or were exposed to ionic dissolution products of the BG scaffolds, kept in permeable cell culture inserts (2D or indirect culture). Though we did not observe any direct osteoinduction of BMSCs by alkaline phosphatase (ALP) assay or by PCR, there was increased vascular endothelial growth factor (VEGF) expression, observed by PCR and ELISA assays. Additionally, the scaffolds showed no toxicity to BMSCs and there were healthy live cells found throughout the scaffold. To analyze further the reasons behind the increased VEGF expression and to exploit the benefits of the finding, we used the indirect method with HDMECs in culture plastic and Cu^2+^-doped BG scaffolds with or without BMSCs in cell culture inserts. There was clear observation of increased endothelial markers by both FACS analysis and acetylated LDL (acLDL) uptake assay. Only in presence of Cu^2+^-doped BG scaffolds with BMSCs, a high VEGF secretion was demonstrated by ELISA; and typical tubular structures were observed in culture plastics. We conclude that Cu^2+^-doped BG scaffolds release Cu^2+^, which in turn act on BMSCs to secrete VEGF. This result is of significance for the application of BG scaffolds in bone tissue engineering approaches.

## Introduction

Bone tissue engineering requires application of suitable biomaterials and cells, which can effectively produce a bone-like tissue matrix in them and, which can substitute the bone defect site [1]. However, the cells cannot survive *in vivo*, when they are placed 200–500 µm from any capillary [2]. Without proper nutrition from a vascular channel, the cells become necrosed and the whole system as a bone replacement product fails to replace the lost tissue. The vascularized bone is crucial for weight-bearing lost bone, substantial bone loss, and for precise healing of the defect site. Therefore, not only the choice of the biomaterial itself, but also the achievement of an effective way to vascularize it, are equally important.

Bioactive glass (BG) is an important biomaterial for scaffold-based bone tissue engineering applications, as it has shown high osteo-inductive and angio-inductive properties compared to other biomaterials [3], [4]. Calcium phosphate-based apatite is produced on the surface of BG in contact with body fluids, and thus BG represents a suitable substrate to promote bone formation both *in vitro* and *in vivo*. In addition, the release of inorganic ions in the media affects the cell behavior for osteogenesis [5], [6].

Though BG scaffolds have been successfully tested for bone defect in small animal models, a patent vascularization is mandatory for the survival of the seeded cells in the scaffold at the defect site. A number of approaches are advocated for the vascularization, including the seeding with endothelial cells (EC), vasculogenic growth factors, and induction of angiogenesis using surgical techniques such as the arterio-venous loop concept [7]–[9]. A novel intelligent approach would be by modification of scaffold components to increase its intrinsic angiogenic potential. As bone is composed of hydroxyapatite (HA) matrix along with a number of microelements, biomolecules, and cells: materials with complex chemical composition and not simple pure materials are more relevant for bone replacement [10]. Usual elements tested as the addendum to bone replacement materials are copper (Cu^2+^), zinc (Zn^2+^), magnesium (Mg^2+^), and strontium (Sr^2+^) [11]–[13]. Among them, Cu^2+^ is considered an important component for osteogenesis and angiogenesis [14] [15].

Compared to growth factor application, the use of inorganic elements (ions) such as Cu^2+^ has multifold advantage. First, they are not degraded by the usual processing of the scaffold and prove stable in harsh conditions involved in scaffold fabrication (e.g. high temperatures). Second, within a limit there is no toxicity as observed with strong bioactive growth factors, and they can be usually excreted through body fluids. Third, inorganic elements are very cost-effective and the additive effect of minimal amounts of growth factors (VEGF, FGF-2) could be highly relevant to the physiological characteristics of the bone environment. Therefore, there is a current need to generate such novel intelligent biomaterials with added inorganic ions to mimic the bone micro-milieu environment.

The aim of the study is two-fold: firstly to investigate the additional osteo- or angio-inductive role of Cu^2+^ by considering copper-doped BG-2D (two dimensional) scaffolds. Secondly to assess the effect of released Cu^2+^ from Cu^2+^-doped BG scaffolds on phenotype and functionality of 2D cultured human dermal microvascular endothelial cells (HDMEC).

## Materials and Methods

### Scaffold fabrication

The BG with different CuO contents were produced by mixing silicon (Si) oxide, sodium carbonate (Na2CO3), calcium carbonate (CaCO3), tri-calcium phosphate (Ca3(PO4)2) and basic Copper carbonate (CuCO3*Cu(OH)2) as described elsewhere [16]. The raw materials were melted at 1450°C for 45 min. The glass was then milled to the final particles size of d50 = 5 µm. Cu2+ containing BG derived scaffolds were produced using the foam replica technique as initially reported [17]. In this method, a sacrificial template is used, namely polyurethane foam (45 ppi, Recticel, UK), which is immersed in a slurry containing BG particles. In the present investigation, a slurry containing 60 wt.-% BG-particles and 1.1 wt.-% PVA (poly-vinyl alcohol) as the binder was used. After coating, the PU foams were dried at 60°C for 24 h. To densify the struts of the scaffolds the dried bodies were sintered at 1050°C for 2 h. During the sintering heat treatment crystallization of the silicate structure occurred, as discussed elsewhere [16] and the scaffolds achieved suitable mechanical stability to be handled for cell culture studies. Cu^2+^ contents of 0.1 wt% and 1 wt% were assessed using plain 45S5 BG scaffolds as control material.

### Common methods for the experiments

#### a. Bone-marrow mesenchymal stem cell (BMSC) procurement and culture

Human BMSCs were purchased from PromoCell GmbH, Heidelberg, Germany at passage two. They were cultured in flasks (COSTAR, Cambridge, USA) by using MSC growth medium with supplemented cytokines (PromoCell GmbH, Heidelberg, Germany), in an incubator with a humidified atmosphere maintained at 37°C and 5% CO_2_. The media were changed twice weekly. At 80–90% confluence, cells were trypsinized (Trypsin/EDTA, PAA, Pasching, Austria) and cultured further as per the recommendation of the company. They were sub-cultured at 80% confluence until passage 5. For all the experimental protocols, passage 5 cells were trypsinized and used directly. Tri-lineage differentiation (osteogenic, adipogenic, chondrogenic) was demonstrated in 2D conditions for BMSCs prior to their use (Figure S1). The cells are found to be highly positive for CD73, CD90, CD105 and negative for CD14, CD20, CD34, CD45 after passage 5 [10] using flow cytometry (FACS Calibur, BD biosciences, Heidelberg, Germany) using a human MSC phenotyping kit (Miltenyi Biotec GmbH, Bergisch Gladbach, Germany), as recommended by the International Society for Cellular Therapy (ISCT).

The scaffold-cell constructs and MSCs in 2D were cultured in basal media (DMEM/Ham’s F-12 (1∶1) with 10% fetal calf serum, 2 mg/L of L-glutamine -all purchased from Biochrom AG, Berlin, Germany).

#### b. Human dermal microvascular endothelial cells (HDMEC) procurement and culture

HDMEC and the specialized endothelial cell growth media (EGM) were obtained from PromoCell GmbH, Heidelberg, Germany. Only passage 5 cells were used for this experiment.

#### c. Copper quantification

Cu^2+^ concentration in the cell culture medium was quantified by inductively coupled plasma - mass spectroscopy (ICP-MS, Varian, Darmstadt, Germany) against standard solutions of 50 and 100 µg/l.

#### d. Experimental design

The scaffold samples were divided into three groups based on the amount of copper in them (Table 1): group A for pure BG scaffolds, group B for 0.1% copper-doped BG, and group C for 1% copper-doped BG scaffolds. Similar groupings were done both for the 2D (indirect) and 3-dimensional (3D, direct seeding) experiments.

**Table 1:** Groups and study design for experiments with bone-marrow derived stem cells (BMSCs) in Bioglass scaffolds.

Method	Scaffoldtype	Groupnames	AlamarBlue	ALP	Live-deadassay	Actinstaining	SEM	RNAcollection	TimePoints
Indirect- 2D	BG	A-2D	(3)	4	2	0	0	4	Week 2, Week 4
	BG with0.1% Cu^2+^	B-2D	(3)	4	2	0	0	4	Week 2, Week 4
	BG with1% Cu^2+^	C-2D	(3)	4	2	0	0	4	Week 2, Week 4
Direct- 3D	BG	A	(3)	4	2	2	2	4	Week 2, Week 4
	BG with0.1% Cu^2+^	B	(3)	4	2	2	2	4	Week 2, Week 4
	BG with1% Cu^2+^	C	(3)	4	2	2	2	4	Week 2, Week 4

Three types of scaffolds were used: only bioactive glass (BG)(Group A), BG with 0.1% Cu^2+^ (Group B), or BG with 1% Cu^2+^ (Group C). The BMSCs are either places on culture plastic keeping the scaffolds in inserts (Indirect) or directly seeded on the scaffolds (Direct). For each time point, 14 scaffolds were used as per the distribution shown below.

Only passage 5 cells were trypsinized and used directly. In 2D experiments, the scaffolds were suspended by cell culture inserts (BD Falcon, Durham, NC, USA) and MSCs are seeded at approximately 20,000 cells per well of 12-well culture plate (BD Falcon, Durham, NC, USA). In 3D experiments, all scaffolds were seeded with 10^5^ cells per scaffold and cultured with basal culture media without any supplements for 4 weeks. The samples were evaluated after 2 and 4 weeks.

To investigate the effect of BMSC-seeded scaffolds on ECs, passage 5 HDMECs (10^5^ cells) were seeded into each well of 12-well culture plastics (BD Falcon, Durham, NC, USA). BG or BG with 1% Cu^2+^ scaffolds were either seeded with BMSCs or kept unseeded. The constructs were taken in cell culture inserts (BD Falcon, Durham, NC, USA) and placed above the HDMECs. For this, we used a cocktail media consisting of one part of basal media and one part of EGM without added VEGF component. HDMECs cultured in only cocktail media served as control. The detail groupings and the analysis experiments were shown in Table 2. The cultured media were later collected to estimate VEGF by ELISA and Cu^2+^ quantification.

**Table 2:** Groups and study design for experiments with human dermal microvascular endothelial cells (EC) and bone-marrow derived stem cells (BMSCs) co-culture in bioactive glass (BG) scaffolds.

Scaffoldtype	Cells inscaffold	Groupnames	Microscopy	acLDL uptakeassay	Tubeformation assay	FACSanalysis	TimePoints
–	–	I	(All)	2	2	6	Week 1, Week 2
BG	–	II	(All)	2	2	6	Week 1, Week 2
BG with 1% Cu^2+^	–	III	(All)	2	2	6	Week 1, Week 2
BG	BMSCs	IV	(All)	2	2	6	Week 1, Week 2
BG with 1% Cu^2+^	BMSCs	V	(All)	2	2	6	Week 1, Week 2

The ECs were always seeded on bottom, while the scaffolds with or without BMSCs are in the cell culture insert. For this experiment, we used a cocktail media consisting of one part of basal culture media and one part of endothelial growth media (EGM) without added VEGF component. For each type, 10 wells were used as per the distribution shown below. The collected media were used to estimate VEGF by ELISA and Cu^2+^ content.

### Evaluation techniques of 2D or 3D experiments with mesenchymal stem cells

#### a. AlamarBlue assay

Each week, the cell-seeded scaffolds or the cells in 12-well plates were analyzed by alamarBlue (Biosource Int., Camarillo, CA, USA) assay, as described previously by our group [18]. The absorbance of the reduced dye was measured by a plate reader (SPECTRAmax 190, Molecular Devices, Sunnyvale, CA, USA) and was subsequently calculated as advised by the manufacturer.

#### b. Alkaline phosphatase (ALP) assay

To analyze the ALP content, the samples were washed with 1x PBS and then treated by lysis buffer (10 mM Tris -pH 7.0, 1 mM EDTA, and 0.2% v/v triton X-100; all from Sigma Aldrich GmbH, Steinheim, Germany). The ALP content was assessed by the measurement of the colored complex produced by the hydrolysis of para-nitrophenyl phosphate (p-NPP) (Sigma Aldrich GmbH, Steinheim, Germany), as described previously [18].

#### c. FDA/PI staining for live-dead assay

The cell-seeded scaffolds were washed with 1×PBS and incubated with 2 µg/ml fluorescein diacetate (FDA) (Molecular Probes Inc., Eugene, US) in 1× PBS, for 15 min at 37°C. They were then gently rinsed twice in 1x PBS and placed in 20 µg/ml propidium iodide (PI) solution (Invitrogen GmbH, Karlsruhe, Germany) for two minutes at room temperature. After thorough rinsing in 1x PBS, the specimens were kept in PBS and viewed under a fluorescent microscope (Axiovert 25, Carl-Zeiss AG, Goettingen, Germany). The viable-cell cytoplasms were labeled green, while non-viable cell nuclei were labeled red.

#### d. Scanning electron microscopic (SEM) analysis

After cell culture experiments, the scaffolds were washed with PBS, fixed with a solution containing 3 vol.% glutaraldehyde (Sigma, Germany) and 3 vol.% paraformaldehyde (Sigma, Germany) in 0.2 M sodium cacodylate buffer (pH 7.4) and finally rinsed three times with PBS. All samples were dehydrated in a graded ethanol series (30, 50, 75, 90, 95 and 99.8 vol. %). Samples were maintained at 99.8 vol. % ethanol and critical-point dried. The samples were sputtered with gold, prior to analysis by SEM (ESEM, Quanta 200, FEI, Netherlands).

#### e. Real time RT-PCR

Total RNA was isolated from the 2D cultured MSCs (n = 4) using TRIzol Reagent (Invitrogen, Carlsbad, CA, US) followed by RNeasy Mini Kit (Qiagen, Hilden, Germany) as described previously [18]. Total RNA was converted to cDNA (QuantiTect reverse transcription kit, Qiagen, Hilden, Germany). The amount of cDNA corresponding to 20 ng of total RNA was then analyzed by semi-quantitative real-time PCR (iQ SYBR green, Bio-Rad, Munich, Germany) for selected genes with primers as shown in Table 3 (CFX 96 real time systems, Bio-Rad, Munich, Germany). The gene expressions were normalized to internal GAPDH expression, and the relative fold change was expressed by comparing to that of the group A-2D at week 2.

**Table 3:** The primers used for real time RT-PCR.

Gene	Forward primer	Reverse primer
GAPDH	ATCAAGTGGGGCGATGCTGG	CCATGACGAACATGGGGGCA
RUNX-2	TTACCCCTCCTACCTGAGCCAG	TTCTGAAGCACCTGAAATGCGC
VEGF	AGGAGGAGGGCAGAATCATCA	CTCGATTGGATGGCAGTAGCT

#### f. Cell spreading assay by fluorescent staining of actin

At 2 and 4 weeks, the cell spreading was observed by staining of the cell actin content by a dye (Alexa Fluor 488 Phalloidin, Life Technologies GmbH, Darmstadt, Germany) at 5 units/ml concentration to see the cell spreading on the BG scaffold. The nuclei of the cells are counterstained by 300 nM DAPI (SelectFX, Life Technologies GmbH, Darmstadt, Germany).

### Evaluation techniques of 2D experiments with HDMEC

#### a. Flow Cytometry analysis

The human EC surface markers (CD31, vWF, VEGFR2) were stained at 5×10^4^ cells for each antigen. CD31 was stained by mouse anti-human CD31-Biotin followed by a second staining step with Streptavidin PerCP-eFluor 710 (both from eBioscience, San Diego, CA, USA). Von Willebrand Factor (vWF) was stained by sheep anti-human vWF-FITC (Abcam, Cambridge, UK). Vascular Endothelial Growth Factor Receptor-2 (VEGFR2) was stained by mouse anti-human CD309 (VEGFR2)-Alexa Fluor 647 (Biolegend, San Diego, CA, USA). All staining steps were performed on ice for 30 minutes in dark. Stained cells were analyzed by FACS-Calibur (BD Biosciences, San Diego, CA, USA) and Cell Quest software (Beckton Dickinson, Heidelberg, Germany). Raw data were analysed by FlowJo software (Tree Star, Inc., Ashland, OR, USA).

#### b. Matrigel 2D assay

For analysis of capillary tube formation, 75 µl of Matrigel (Becton Dickinson, Heidelberg, Germany), an extracellular mouse sarcoma matrix known to produce pro-angiogenic stimulus both in vitro and in vivo, was pipetted into each well of a 96-well plate (Falcon, Heidelberg, Germany) and incubated at 37°C for 60 minutes. HDMECs were harvested at week 1 or week 2 and suspended at 50,000 cells per 150 µl of EGM MV2 media. 150 µl of this media were added to the Matrigel coated 96-well plates and incubated for 24 h at 37°C. Capillary tube formation on Matrigel was observed at the end under an inverted Leica DMIL microscope and photos were taken using the Leica application suite software (Leica GmbH, Wetzlar, Germany).

#### c. Alexa Fluor 488-Ac-LDL-staining

At end time points, the media were removed and the cells were washed once with PBS to remove non-adherent cells. The wells were incubated with 2.5 µg/ml ac-LDL-Alexa Fluor 488 (Life Technologies GmbH, Darmstadt, Germany) for 4 h at 37°C. Afterwards cells were washed twice with PBS and further analyzed by a fluorescence microscope (Olympus iX81, Centre Valley, PA, USA.) concerning successful uptake of the dye.

#### d. VEGF quantification ELISA

At each time point, the cultured media were collected from 3 different samples, pooled together, labeled and frozen at −20°C. At the end of all experiments, the frozen media were thawed overnight at 4°C and the VEGF content of the media was quantified by using an ELISA kit (Thermo Fisher Scientific GmbH, Schwerte, Germany).

### Statistical analysis

All statistical comparisons were performed using a one-way ANOVA test followed by Bonferroni’s post-test (Sigmastat v3.5, Chicago, IL) considering a significant difference at the 95% confidence interval. The results were expressed as means ± standard deviation and considered significant at p<0.05 level. For all pair-wise comparisons of quantitative results, the Student’s t-test was used with a confidence level of 95% (p<0.05). In all figures, the significant results among different samples (time points) in the same group is marked by ‡ and among same time point samples in different groups is marked by *.

## Results

The Cu^2+^-45S5 derived scaffolds exhibit high porosity of ∼90% and highly interconnected pore system with pore sizes ranging from 200 to 450 µm. The structural properties and the acellular bioactivity of the scaffolds used in this study have been investigated previously [16]. The summary of different assays obtained by different tests are summarized in Table 4.

**Table 4:** Summary of different assays performed in the experiment and the results obtained in each of the groups.

Experimental set-up	Tests	Gr. A	Gr. B	Gr. C	MSC only	
2D or indirect culture	Alamar blue, ALP assay, RT-PCR of osteogenic markers	No difference				
	RT-PCR of VEGF	No difference		Increased	No difference	
3D or direct culture	Alamar blue, ALP assay, SEM or cell attachment	No difference				
	Cu^2+^ quantification	–	+	+++	–	
		Group I	Group II	Group III	Group IV	Group V
2D indirect assay with both MSCs and ECs	Matrigel assay with the end-of-time cells	All shows positive tube formation.				
	Light microscopy					Tube-like formation
	Flow cytometry for VWF				Wk 1∶77%; Wk 2∶60.3%	Wk 1∶92.5%, Wk 2∶95.1%
	Flow cytometry for KDR				Wk 1∶34%, Wk 2∶16.5%	Wk 1∶66%, Wk 2∶47%
	Flow cytometry for CD 31				Wk 1∶90%, Wk 2∶97.3%	Wk 1∶95.5%, Wk 2∶98%
	Acetylated LDL uptake	+	+	+	+	+
	VEGF in media	–	–	–	++	++
	Cu^2+^ quantification	–	–	+	–	+

(Kindly refer the text for details).

### Indirect-2D analysis with MSCs

**Figure 1:**
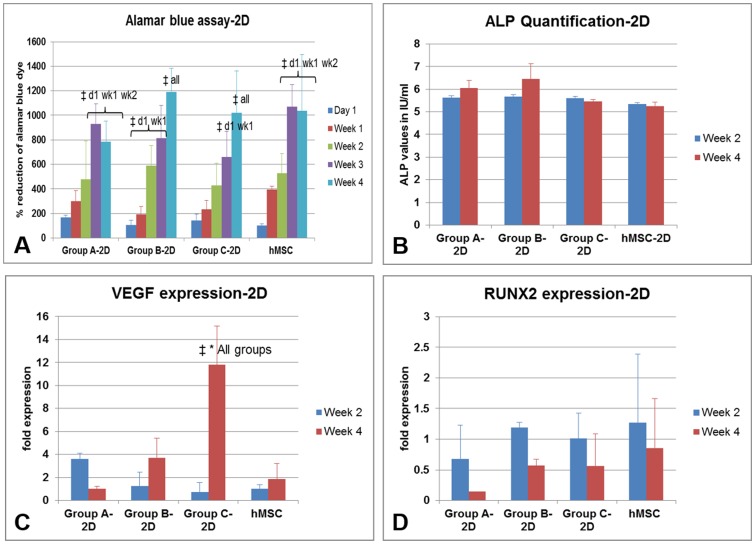
The MSCs in 2D were analyzed by (A) alamar blue dye reduction assay, (B) alkaline phosphatase quantification; (C) real time PCR for VEGF expression and (D) RUNX2 expression. The scaffolds were suspended in cell culture inserts as described in materials and method section. The significant results among different samples (time points) in same group is marked by ‡ and among same time point samples in different groups is marked by *.

The alamarBlue reduction test shows progressive increased reduction of the dye from day 1 to week 4 (Figure 1A). At each time point the dye reduction is not different among other groups and only MSCs. However, the dye reduction values at week 3 and week 4 are significantly higher than day 1 and week 1 values in most of the groups (Figure 1A). There is a basal expression of ALP in all cells without any difference among each other and compared to MSC-only samples (Figure 1B). The real time PCR was expressed as the fold expression of the gene of interest compared to the expression in MSC-only samples at week 2, all normalized to their Glyceraldehyde 3-phosphate dehydrogenase (GAPDH) expression. Interestingly, only group C-2D samples show multifold higher VEGF expression compared to any other sample (Figure 1C). The osteogenic gene RUNX-2 expression was not significantly different among each other (Figure 1D).

### Direct-3D analysis of bioglass-MSC constructs

**Figure 2:**
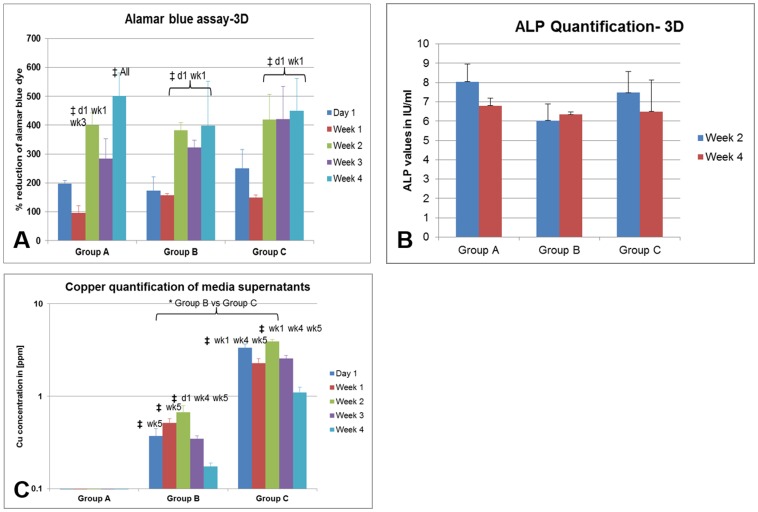
The MSCs in bioactive glass scaffolds were analyzed for (A) alamar blue dye reduction assay, (B) alkaline phosphatase quantification, and (C) copper measurement of the supernatant media. The significant results among different samples (time points) in same group is marked by ‡ and among same time point samples in different groups is marked by *.

The alamarBlue reduction test shows a sudden increased reduction of the dye at week 2, which remains stable up to week 4 (Figure 2A). At each time point the dye reduction is not different among all groups. However, all groups show a significant reduction at week 4 compared to their day 1 values. Similar to 2D experiment, there is a basal expression of ALP in all cells without any difference among all groups (Figure 2B). As demonstrated in the 2D experiments, copper-containing specimens displayed increased VEGF expression (Figure S2). Additionally, the Cu^2+^ estimated from all respective media shows significantly increased values in group C than group B and increased values in group B than group A samples (Figure 2C). In groups B and C, the estimated Cu^2+^ is significantly increased at week 3, though maintaining high values throughout all time points.

**Figure 3:**
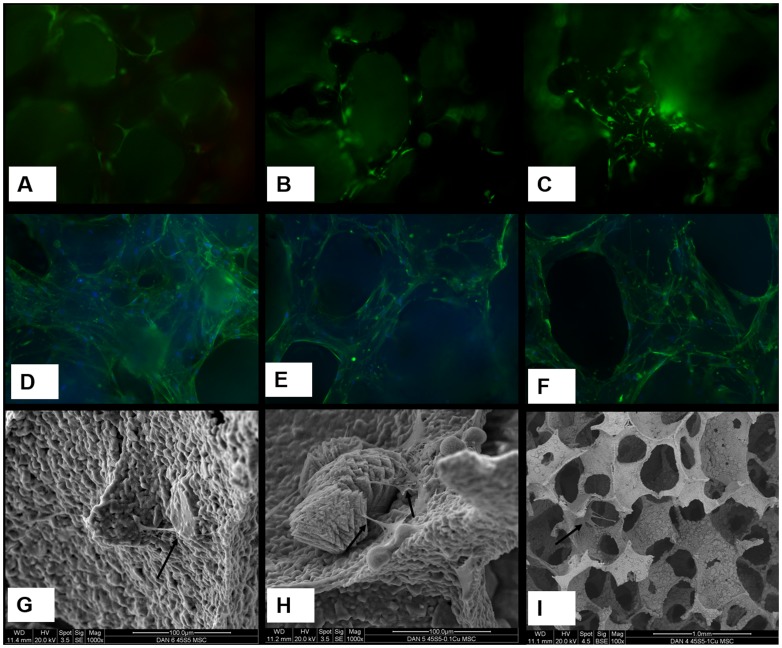
Fluorescent staining of live cells as green and dead cells as red (A, B, C) and the cyto-skeletal staining of actin showing the cell spreading (D, E, F), and scanning electron microscope pictures (G, H, I) for group A = only bioglass scaffolds (A, D, G), group B = scaffolds with 0.1% Cu^2+^ (B, E, H), and group C = scaffolds with 1% Cu^2+^ (C, F, I). All samples are shown at week 4, though similar pictures were observed also at week 2. In SEM pictures, the cells are shown by arrows. Note the magnification is different in (I).

The live-dead assay shows all cells are alive without any significant dead cells attached to the scaffold (Figure 3 A, B, C). The cells spread well and closely attach to the struts of BG scaffolds as shown by actin staining (Figure 3 D, E, F). The cells were directly observed by SEM analysis attaching nicely on the scaffolds (Figure 3 G, H, I).

### EC analysis under the influence of Cu^2+^-45S5 scaffolds

**Figure 4:**
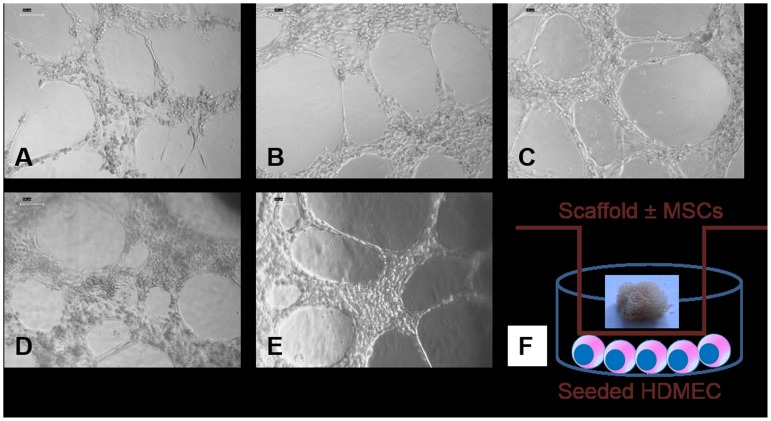
At the end of week 2, HDMECs were trypsinized and assayed for tube formation in Matrigel. (A) group I, (B) group II, (C) group III, (D) group IV, and (E) group V. Similar pictures were observed also at week 1. (F) Schematic diagram showing the scaffold seeded with cells in culture inserts and one layer of HDMECs at the bottom as in groups IV and V.

Under the influence of different constructs as depicted in Table 2, the ECs were observed to retain most of their phenotype. They were observed to produce the typical EC property forming tubes in Matrigel, both at week 1 and week 2 (Figure 4).

**Figure 5:**
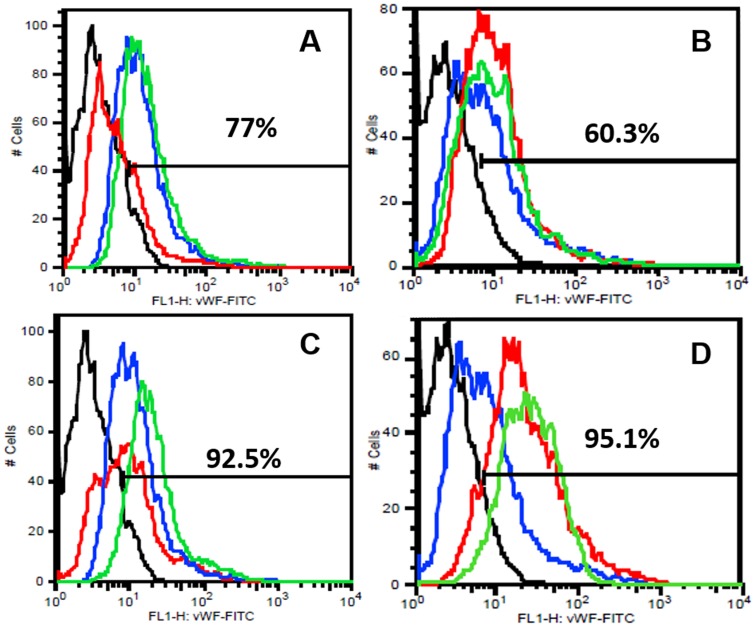
FACS analysis for vWF antigen on surface of HDMECs at week 1 (A,C) and week 2 (B, D). The colors are depicted as black: negative control; blue: cells in cocktail media = group I; red: group II (A, B) or, group III (C, D); green: group IV (A, B) or, group V (C, D). The percentages of total cells positive for the dye in group IV (MSC-bioglass) are indicated in A and B; those in group V (MSC-Cu-bioglass) are in C and D.

**Figure 6:**
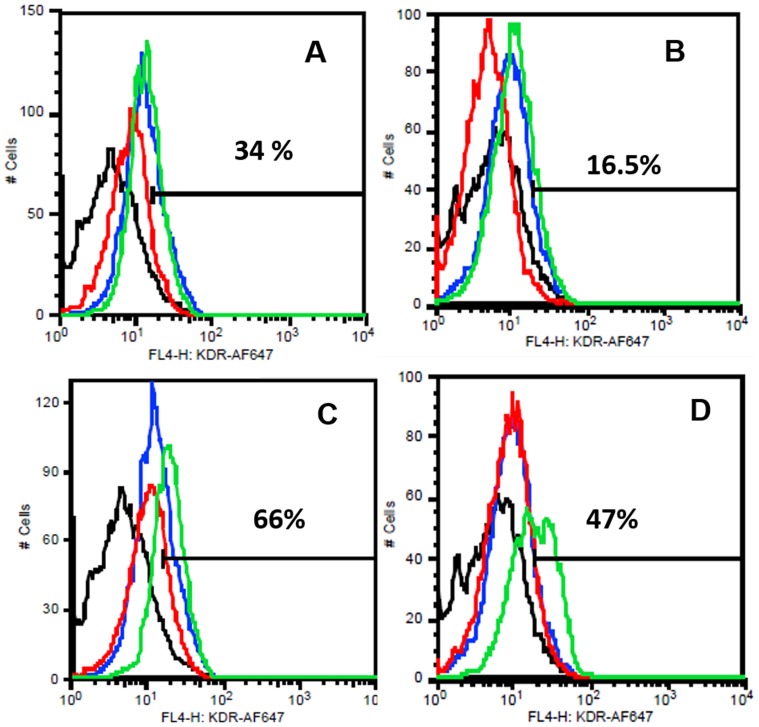
FACS analysis for VEGFR2 on the surface of HDMECs at week 1 (A,C) and week 2 (B, D). The colors are depicted as black: negative control; blue: cells in cocktail media = group I; red: group II (A, B) or, group III (C, D); green: group IV (A, B) or, group V (C, D). The percentages of total cells positive for the dye in group IV (MSC-bioglass) are indicated in A and B; those in group V (MSC-Cu-bioglass) are in C and D.

**Figure 7:**
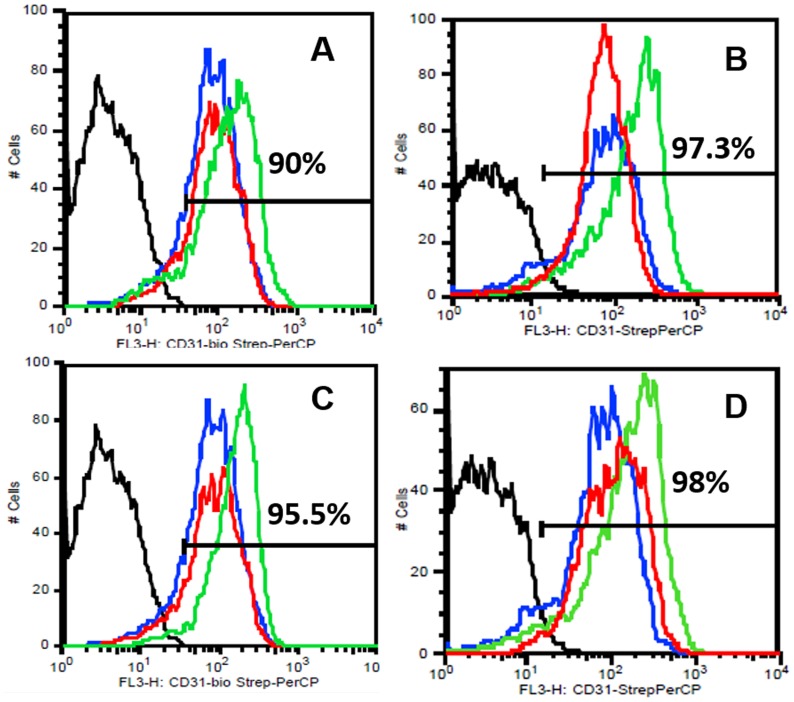
FACS analysis for CD 31 on the surface of HDMECs at week 1 (A,C) and week 2 (B, D). The colors are depicted as black: negative control; blue: cells in cocktail media = group I; red: group II (A, B) or, group III (C, D); green: group IV (A, B) or, group V (C, D). The percentages of total cells positive for the dye in group IV (MSC-bioglass) are indicated in A and B; those in group V (MSC-Cu-bioglass) are in C and D.

To analyze their detailed EC phenotype the flow cytometric analyses were done for different EC markers from pooled HDMECs. It was observed under the influence of BG-MSC (group IV) that, around 77% and 60.3% cells were positive for vWF at week 1 and week 2 respectively. However, the values were 92.5% and 95.1% due to Cu^2+^-BG-MSC (group V) scaffolds (Figure 5). Similarly, it was observed that, under the influence of group IV scaffolds around 34% and 16.5% cells were positive for VEGFR2 at week 1 and week 2 respectively. The values were 66% and 47% due to Cu^2+^-BG-MSC (group V) scaffolds (Figure 6). It was also observed that, under the influence of group IV scaffolds, around 90% and 97.3% cells were positive for CD 31 at week 1 and week 2 respectively. However, the values were 95.5% and 98% due to Cu^2+^-BG-MSC (group V) scaffolds (Figure 7). It is interesting to note that the EC phenotype is better retained in all samples with MSC-added scaffolds than their non-MSC counterparts.

**Figure 8:**
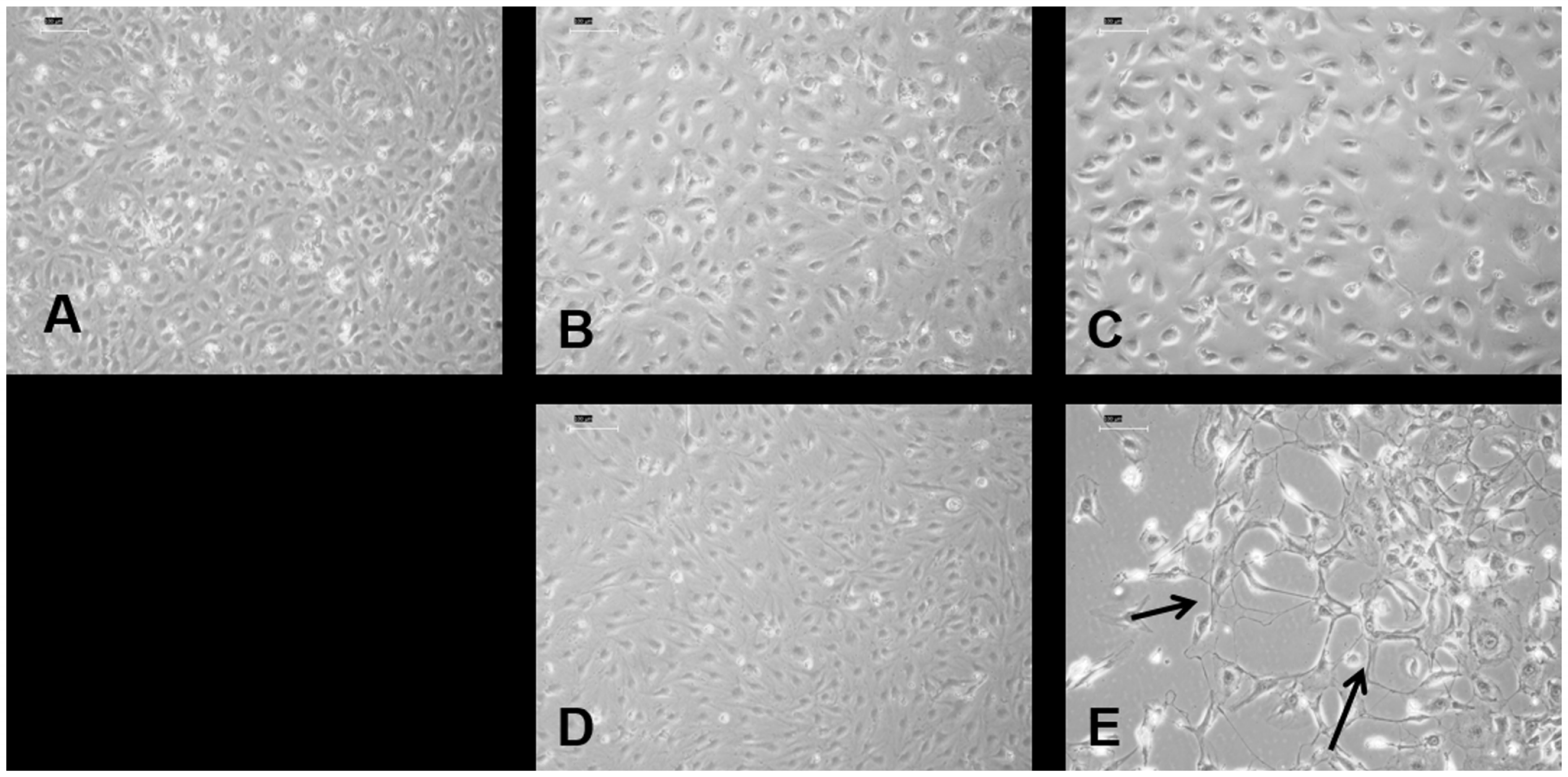
At the end of week 2, light microscopic pictures of HDMECs show tube formation only in group V containing both MSC-seeded Cu^2+^-bioglass inserts (arrows). (A) group I, (B) group II, (C) group III, (D) group IV, and (E) group V.

**Figure 9:**
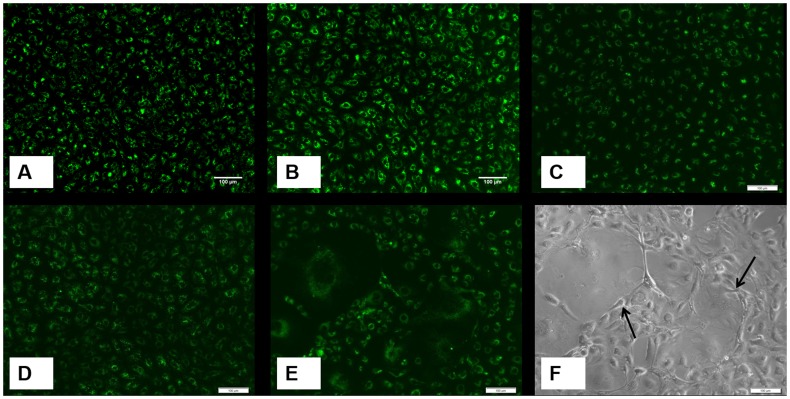
At the end of week 2, acetylated LDL uptake assay is performed in one well seeded with HDMECs. Though all cells uptake the dye, only in group V cells containing MSC-seeded Cu^2+^-bioglass inserts show tube like forms even in 2D cultures (arrows). (A) group I, (B) group II, (C) group III, (D) group IV, and (E) group V. For comparison, a light microscopic picture of the same well is shown in figure F.

**Figure 10:**
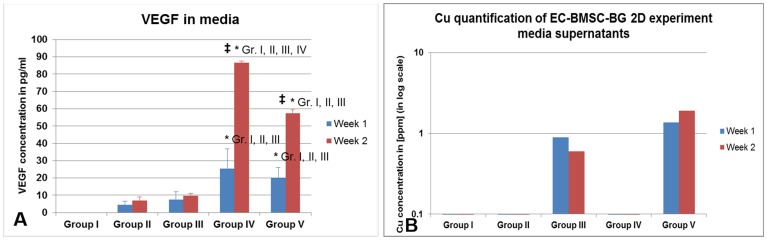
The supernatant media were collected from the EC-MSCs in bioactive glass scaffold experiment and were analyzed for (A) VEGF concentration measured by ELISA and (B) Copper content measured for the same media to correlate with the VEGF data. The significant results among different samples (time points) in same group is marked by ‡ and among same time point samples in different groups is marked by *.

Light microscopic images show some interesting features. Only after week 2, under the effect of Cu^2+^-BG-MSC, the cells were seen to exhibit endothelial tube formation (without the influence of Matrigel), which was absent in all other groups (figure 8E, arrows). The HDMECs are characterized by uptake of acetylated LDL, which was positive in almost all the cells (figure 9). However, only in group V specimens, the endothelial tube-like cell clusters were observed (Figure 9 E, F). At week 2, there is significantly increased amount of VEGF released into the media in group IV and group V (Figure 10). It is interesting to note that VEGF is increasingly secreted in presence of both Cu^2+^ and MSC. However, in presence of only Cu^2+^ there is no increased VEGF secretion (Figure 10A, group III). Without the effect of Cu^2+^, the presence of MSCs in BG is enough to secrete VEGF (Figure 10A, group IV). However, only in the presence of both the EC phenotype is better retained.

## Discussion

We have successfully shown that the Cu^2+^-doped BG scaffolds exhibit no toxicity even up to 1 wt% Cu^2+^ concentration. Both the MSCs and HDMECs were cultured and grown up to week 4 in this scaffold. Moreover, MSCs secrete increased VEGF into media most likely in presence of Cu^2+^. Only in the presence of both Cu^2+^ and MSCs, the HDMECs exhibit strong EC phenotype and the media show high VEGF amount.

In our previous work, we have shown that Cu^2+^-doped 45S5 BG scaffolds exhibit high acellular bioactivity as proven by rapid formation (after 3 days of immersion in simulated body fluid) of a carbonated HA layer on BG scaffolds surface [16]. Moreover, the released Cu^2+^ levels in the simulated body fluid are in the therapeutic range, indicating a potential angiogenic effect of such Cu^2+^-releasing scaffolds. In this study, we expanded the evaluation of Cu^2+^-45S5 BG scaffolds in cell culture in order to confirm potential stimulating effects of Cu^2+^ on MSC and MSC/HDMEC co-culture.

The results show that in the presence of BG and Cu^2+^-BG scaffolds, BMSCs grow abundantly up to week 4. Though there is no significant difference in AlamarBlue reduction value at each time point, there is significantly increased VEGF mRNA expressed in presence of 1% Cu^2+^ doped scaffolds (Figure 1C). Regarding the cell number, vitality, and cell spreading no significant difference was observed among the different Cu^2+^-doped scaffolds (Figure 3). Our observations are in good accordance with the literature: most likely, Cu^2+^ ions stabilize and upregulate hypoxia-inducible factor 1 (HIF-1) [19] which, in turn, regulates the VEGF expression in MSCs [20]. Although the VEGF response was seen at a later time point, the current data cannot be extrapolated for in vivo data, where the cells and scaffolds are applied in 3D environment. This is a limitation of this study.

The possible change of solubility of the BG with incorporation of Cu^2+^ should not lead to major biological effects, based on previous results of their surface reactivity [16]. In particular, the dissolution behavior and the bioactivity of the Cu^2+^-doped BG in comparison with the un-doped 45S5 BG was comprehensively investigated in simulated body fluid [16]. It was found that there was no significant effect due to Cu^2+^ addition (up to 2.5 wt. % CuO) on the reactivity of the un-doped BG, which was measured by the formation of HA on the surface of the material. This fact was confirmed by Fourier transformed infrared spectroscopy (FTIR), scanning electron microscopy (SEM), and X-ray diffraction analysis (XRD). Since HA formation depends on the dissolution of Na, Ca, Si, and P ions from the BG, the formation of similar level of HA directly confirms the fact that Cu^2+^ addition did not affect the surface reactivity of the glass scaffolds. Nevertheless, it is not expected that the dissolution of Si, Na, Ca and P ions from the glass would be affected by the rather low addition of Cu^2+^ (up to 1wt.% in the present study). Moreover, by detailed elemental composition analysis using Particle Induced X-ray Emission/Rutherford Backscattering Spectrometry, it was concluded that the global dissolution of the 45S5 BG is independent of Cu^2+^ doping. No major differences were observed in FT-IR and Raman spectroscopy results confirming that the SiO2 network dominates the behavior of the BG, which was not significantly affected by the Cu^2+^-doping under static conditions, similar to the cell culture conditions of this present study. It was also shown that Si ion release was not affected by Cu^2+^ addition [16].

### Functional role of VEGF in the scaffold-cell constructs

To prove the functional aspects of secreted VEGF, we have tested HDMECs in culture plastics in presence/absence of 45S5- Cu^2+^ BG scaffolds seeded with MSC in permeable cell culture inserts. Interestingly, only in presence of MSCs, we have observed a high VEGF amount in media. The presence of Cu^2+^ alone is not sufficient to make HDMECs secrete VEGF (Figure 10). Additionally, only with MSCs and Cu^2+^-45S5 scaffolds, a higher number of ECs shows positive markers such as CD 31, vWF, and VEGFR2 (KDR, VEGF receptor 2). We have also observed the EC tube formed only in these samples. Though the tube formation in ECs is not typical as found in a strong stimulus medium as Matrigel, we observe this significant morphological response consistently in copper doped scaffolds. They indicate the presence of Cu^2+^ in this system is favorable for the HDMECs for their EC-phenotype, even though the VEGF content in the culture media is comparable for HDMECs seeded in contact with MSCs on 45S5 scaffolds and Cu^2+^-containing 45S5 scaffolds (groups IV and V in Fig. 10A). As our control specimens are undoped 45S5 bioglass scaffolds, it is suggested that the observed effects on cell behavior are based on the presence and effect of the Cu^2+^ ions, and might not be due to any other ions. However, a potential synergetic effect of Cu^2+^ in combination with other relevant ions being released such as Calcium, Phosphorous, and Si should not be ruled out. This implies that in addition to the increased VEGF, there must be other mechanisms by Cu^2+^ leading to proper functional retention of HDMECs. It is most likely the Cu^2+^ acting on MSCs produces such a high VEGF expression. Therefore, we conclude that it is a synergistic action by both Cu^2+^ and MSCs for the retention of functionality of HDMECs. Though Cu^2+^ doped specimens show tube-like formation by ECs, but they could not substitute completely for growth factor added EGM. This is a valid limitation of our study. We assume there might be a beneficial role of Cu^2+^ on ECs, but the EGM might not be completely substituted by copper-doped scaffolds.

### Bio-functional role of Copper on angiogenetic/vasculogenic applications

Cu^2+^ ions regulate EC proliferation, migration, and they can enhance vascular permeability, as shown by Li et al. [21]. Additionally, Cu^2+^ has been shown to activate angiogenic growth factors such as VEGF and FGF (fibroblast growth factor) due to activation of hypoxia-VEGF secretion, MAPK, tyrosine kinase pathways [14], [22]. It has already been reported that VEGF expression can be induced by copper ions and this property may be exploited to accelerate dermal wound healing [14]. However, the VEGF secretion is helpful due to the presence of other cell types such as MSC in our study or keratinocytes, as reported in the literature [14]. Additionally, lysyl oxidase, a Cu^2+^ containing enzyme, cross-links the lysine-derived aldehyde and plays a crucial role in collagen maturation during tissue regeneration [14].

In another study, when 3D printed bioceramic scaffolds were investigated *in vivo* in mice for their angiogenic ability, there were similar results observed with Cu^2+^-loaded scaffolds to those with VEGF-loaded scaffolds [23]. The tissue ingrowth was observed to be dose-related to Cu^2+^ concentration up to a level and additive with VEGF. It was also shown that Cu^2+^ was released during the long degradation process of the scaffold and hence a sustained release was possible, which is similar to the one observed in our study. The burst release of Cu^2+^ might have caused toxicity. VEGF or FGF-2 has been reported to have synergistic effects on the angiogenic effect of CuSO_4_ *in vivo* [24]. Cu^2+^ ions are also involved in the activity of some transcription factors via Hypoxia Inducible Factor-1α and proline hydroxylase, facilitating the release of GFs and cytokines from producing cells [24]. There is also direct evidence of the usefulness of Cu^2+^ in increased flap survival in rats due to enhanced VEGF expression [25]. This action is again due to the action of Cu^2+^ on nearby cells in the random flap in inducing VEGF.

### Additional value of Cu^2+^ in MSC-EC co-culture setting

There is a recent trend of using co-cultures of MSCs and ECs (HDMECs or human umbilical cord derived ECs) in tissue engineering applications [26], [27]. Hereby, the idea is to stimulate MSCs to differentiate in osteoblast lineage, and ECs to induce the necessary vascularization for nutrition supply to MSCs. However, to make this scenario possible, an additional high amount of VEGF is added to make the best use of ECs and facilitate an early vascularization [28]. In our current study, we have shown that Cu^2+^-doped BG scaffolds can act on MSCs to secrete high amount of VEGF. Therefore, such scaffolds could prevent the cost and potential disadvantage of growth factor dependent models. Currently, we are investigating the effect of this scaffold with MSCs *in vivo* for successful bone generation. It was also recently reported that there exists a synergistic effect of Cu^2+^ and Si ions (released from BG) on stimulation of vascularization [29].

### Bio-functional role of Copper on osteogenic applications

The osteoinduction action of Cu^2+^ on MSCs has been also reported [12], [15]. For example, Cu^2+^ was shown to modify both the differentiation and proliferation of BMSCs obtained from post-menopausal women. 50 µM of Cu^2+^ diminished the MSC proliferation but increases their ability to differentiate into both osteo- and adipogenic lineages [12]. In their study, Cu^2+^ caused a two-fold increase in calcium deposition. Interestingly, both 5 and 50 µM of Cu^2+^ induced diminished ALP expression but provoked a shift in its expression to earlier time points [12]. However, in those cases the cells were induced with osteoinducing media and Cu^2+^ showed only an additive action on osteoinduction. Interestingly, in our study we did not find any significant effect of Cu^2+^ on MSC differentiation. The reason could be due to the fact that BG scaffolds have inherent osteoinduction ability [30] and thus Cu^2+^ ions might not lead to enhanced osteoinduction above the intrinsic osteoinductivity of BG scaffolds. In addition, in this study no osteoinduction medium was applied, which has been used in previous studies [12]. In this investigation, we considered only the effect of Cu^2+^ on MSCs without any additional promoting factors in osteoinduction. Supporting our results, previous studies have been reported, where calcium phosphate minerals were used with different trace elements, such as Cu^2+^ and Zn^2+^.It has been shown that Cu^2+^ and Zn^2+^ not only inhibit osteoblast proliferation, but also prevent their differentiation [31]. In addition to Cu^2+^, also cobalt (Co^2+^) has been tested for its angiogenetic properties using Co^2+^ doped BG scaffolds [32].

### Importance of finding of optimum dosage of copper for specific applications

It was shown that the copper content of 24 µg per gm (wt./wt.) in a hydrogel scaffold stimulated EC growth in cultures and 75 µg per gm wt./wt. in scaffold had an angiogenic potential upon implantation [23]. The lower limit was 56 ng per scaffold, facilitating angiogenesis during tissue ingrowth; whereas a 10-fold increase in Cu^2+^ dose (560 ng per scaffold) caused enhanced wound healing. The limit for would healing was successfully lowered when VEGF was combined to copper sulfate [23]. In a fibrin glue system, a dose-dependent increase of the extension of ECs into tiny cord and tube-like structures was observed, which was measured to reach a peak at 50 ng/ml. The observed bell-shaped curve response at higher doses of CuSO_4_ was explained considering that, with increased Cu^2+^, there was also an increase in reactive oxygen species, therefore, leading to cytotoxicity [24].

A similar study to ours was reported by Wu et al. [15], in which Cu^2+^-doped BG scaffolds as multifunctional scaffolds were investigated, having combined angiogenic, osteo-stimulative, and antibacterial properties. It was shown that both the scaffolds and their ionic extracts could stimulate HIF-1α and VEGF expression. Though they have reported that Cu^2+^ has added osteogenic ability only at very high concentrations, the ALP concentration did not show significant difference among cells in different Cu^2+^-BG scaffolds [33]. Similarly only with very high Cu^2+^ concentrations the osteogenic genes are multi-fold expressed, which was not seen at lower doses. The authors explained the results of increased angiogenesis attributed to hypoxia-like tissue reaction. However, it was shown that with the highest Cu^2+^ concentration, there was increased cytotoxicity and hence decreased cell viability [33]. In the present study, it was considered impractical to use high Cu^2+^ concentrations and the study was limited to 1% Cu^2+^ in BG scaffolds. The present results showed the additional effect of Cu^2+^ on MSC to secrete VEGF and it was expanded to investigate also the effect on EC phenotype and functions. However, we found our Cu^2+^ concentration is different from those already reported. As we specifically measured the free released elemental Cu^2+^ ions in the media by highly sensitive inductively coupled plasma-mass spectroscopy and used a different manufacturing process, we assume these variations and the special composition of our material affect the released pattern of copper ions differently.

### Possible cytotoxicity of higher dosage of Cupper

In another study, Copper (both cuprous and cupric) was found to be cytotoxic [34]. This cytotoxic effect was not due to apoptosis, but by necrotic cell death. Therefore, the minimal dosage was investigated here to determine the effect without entering the toxic concentration level. We have found that MSCs at 1% Cu^2+^ grow normally up to 4 weeks without any significant dead cells. Hydrogen peroxide toxicity in osteoblasts has been reported to be significantly enhanced in the presence of metal ions such as Cu^2+^ ions. They produce the toxic hydroxyl radical by specific reaction, which causes cell necrosis [35].

### Advantage of copper over other modalities to induce angiogenesis

Copper doped BG scaffolds pose a special advantage over other modalities to enhance angiogenesis. Though the growth factors such as VEGF induce strong angiogenesis, incorporating them before scaffold processing could reduce their potency, whereas applying them at end stage could produce non-desired side effects, including cancer outside the scaffold area [36]. In addition, these scaffolds could be highly expensive. EC is applied with MSCs in a number of other studies. Though the reports point out vascularization with these two types of cells, they usually require added growth factors for proper effectiveness of the constructs. Cu^2+^ could make the best use of the two-cell system by producing VEGF from MSCs as shown in this study [14], [21]. In addition, the whole system is cost-effective for tissue engineering applications.

In this study, we have shown that the Cu^2+^ ions in BG scaffold act on MSCs to have high VEGF secretion into the media. The secreted VEGF and the Cu^2+^ ions have influence on EC functional properties. The HDMECs retained their surface antigens and produced tube-like structure even in cell culture only in presence of Cu^2+^ and MSCs. Thus, by stimulating MSCs to release increased VEGF, which in turn stimulated ECs, the current Cu^2+^-BG scaffolds act as an “indirect” angiogenic growth factor delivery system [37]. This indirect approach is advantageous, since it enables controlled VEGF release mediated by cells, which can be adopted physiologically to the local needed conditions, avoiding growth factor overdose. This knowledge could be used in EC-MSC co-culture systems with this Cu^2+^-doped BG scaffolds for successful application in bone regenerative medicine.

## Conclusions

We have reported the usefulness of Cu^2+^ ions in a minimal amount to the benefit of available cells, especially MSCs for bone tissue engineering application. The attractiveness of BG scaffolds for bone tissue engineering can be enhanced by the addition of Cu^2+^ and in the presence of other cell types to harness the VEGF production machinery of cells in a cost-effective and relevant manner for proper angiogenesis *in vivo*. Hence, Cu^2+^-doped BG scaffolds in combination with MSCs are superior candidates for bone tissue engineering application with enhanced angiogenic potential. The combined approach should be further evaluated for *in vivo* cell-based tissue engineering applications.

## Supporting Information

Figure S1 **BMSCs are seeded in two-dimensional way in culture plastics.** They are grown until 70% confluency and later differentiated into different lineages. After osteo-induction for 4 weeks, the cells are stained by alkaline phosphatase (A); after adipo-induction for 2 weeks, the cells are stained by oil red O (B); after chondrogenic induction for 2 weeks in a pellet culture, they are stained by alcian blue (C).
(TIF)

Figure S2 **The supernatant media were collected from the MSC-BG (bio-active glass) constructs and were analyzed for VEGF concentration measured by ELISA.** The significant results among different samples (time points) in same group is marked by ‡ and among same time point samples in different groups is marked by *. There was no adequate samples from Group C, for which the data for Group C are not presented.
(TIF)

## References

[1] Horii A, Wang X, Gelain F, Zhang S (2007) Biological Designer Self-Assembling Peptide Nanofiber Scaffolds Significantly Enhance Osteoblast Proliferation, Differentiation and 3-D Migration. PLoS ONE 2. 10.1371/journal.pone.0000190 PMC1784071 17285144

[2] Rath SN, Arkudas A, Lam CXF, Olkowski R, Polykandroitis E, et al (2012) Development of a pre-vascularized 3D scaffold-hydrogel composite graft using an arterio-venous loop for tissue engineering applications. Journal of Biomaterials Applications 27:277–289. 21680609 10.1177/0885328211402243

[3] Gerhardt LC, Widdows KL, Erol MM, Burch CW, Sanz-Herrera JA, et al (2011) The pro-angiogenic properties of multi-functional bioactive glass composite scaffolds. Biomaterials 32: 4096–4108. 21411138 10.1016/j.biomaterials.2011.02.032

[4] Varanasi VG, Owyoung JB, Saiz E, Marshall SJ, Marshall GW, et al (2011) The ionic products of bioactive glass particle dissolution enhance periodontal ligament fibroblast osteocalcin expression and enhance early mineralized tissue development. Journal of Biomedical Materials Research Part A 98:177–184. 21548068 10.1002/jbm.a.33102 PMC6500091

[5] Liu Q, Cen L, Yin S, Chen L, Liu G, et al (2008) A comparative study of proliferation and osteogenic differentiation of adipose-derived stem cells on akermanite and β-TCP ceramics. Biomaterials 29:4792–4799. 18823660 10.1016/j.biomaterials.2008.08.039

[6] Hoppe A, Guldal NS, Boccaccini AR (2011) A review of the biological response to ionic dissolution products from bioactive glasses and glass-ceramics. Biomaterials. 10.1016/j.biomaterials.2011.01.004 21292319

[7] Arkudas A, Balzer A, Buehrer G, Arnold I, Hoppe A, et al (2013) Evaluation of Angiogenesis of Bioactive Glass in the Arteriovenous Loop Model. Tissue Engineering Part C: Methods 19:479–486. 23189952 10.1089/ten.tec.2012.0572 PMC3629783

[8] Schmidt VJ, Hilgert JG, Covi JM, Weis C, Wietbrock JO, et al (2013) High Flow Conditions Increase Connexin43 Expression in a Rat Arteriovenous and Angioinductive Loop Model. PLoS ONE 8:e78782. 24236049 10.1371/journal.pone.0078782 PMC3827249

[9] d’Angelo I, Oliviero O, Ungaro F, Quaglia F, Netti PA (2013) Engineering strategies to control VEGF stability and levels in a collagen matrix for angiogenesis: the role of heparin sodium salt and the PLGA-based microsphere approach. Acta Biomaterialia 9:7389–7398. 23523534 10.1016/j.actbio.2013.03.013

[10] Rath SN, Nooeaid P, Arkudas A, Beier JP, Strobel LA, et al. (2013) Adipose- and bone marrow-derived mesenchymal stem cells display different osteogenic differentiation patterns in 3D bioactive glass-based scaffolds. Journal of Tissue Engineering and Regenerative Medicine DOI: doi:10.1002/term.1849.. 24357645

[11] Zreiqat H, Ramaswamy Y, Wu C, Paschalidis A, Lu Z, et al (2010) The incorporation of strontium and zinc into a calcium–silicon ceramic for bone tissue engineering. Biomaterials 31:3175–3184. 20117832 10.1016/j.biomaterials.2010.01.024

[12] Rodríguez JP, Rios S, Gonzalez M (2002) Modulation of the proliferation and differentiation of human mesenchymal stem cells by copper. Journal of Cellular Biochemistry 85:92–100. 11891853

[13] Li J, Song Y, Zhang S, Zhao C, Zhang F, et al (2010) In vitro responses of human bone marrow stromal cells to a fluoridated hydroxyapatite coated biodegradable Mg-Zn alloy. Biomaterials 31:5782–5788. 20452668 10.1016/j.biomaterials.2010.04.023

[14] Sen CK, Khanna S, Venojarvi M, Trikha P, Ellison EC, et al (2002) Copper-induced vascular endothelial growth factor expression and wound healing. American Journal of Physiology-Heart and Circulatory Physiology 282:H1821–H1827. 11959648 10.1152/ajpheart.01015.2001

[15] Wu C, Zhou Y, Xu M, Han P, Chen L, et al (2013) Copper-containing mesoporous bioactive glass scaffolds with multifunctional properties of angiogenesis capacity, osteostimulation and antibacterial activity. Biomaterials 34:422–433. 23083929 10.1016/j.biomaterials.2012.09.066

[16] Hoppe A, Meszaros R, Stähli C, Romeis S, Schmidt J, et al (2013) In vitro reactivity of Cu doped 45S5 Bioglass derived scaffolds for bone tissue engineering. Journal of Materials Chemistry B 1:5659–5674. 10.1039/c3tb21007c 32261190

[17] Chen QZ, Thompson ID, Boccaccini AR (2006) 45S5 Bioglass-derived glass-ceramic scaffolds for bone tissue engineering. Biomaterials 27:2414–2425. 16336997 10.1016/j.biomaterials.2005.11.025

[18] Rath SN, Strobel LA, Arkudas A, Beier JP, Maier AK, et al (2012) Osteoinduction and survival of osteoblasts and bone-marrow stromal cells in 3D biphasic calcium phosphate scaffolds under static and dynamic culture conditions. Journal of Cellular and Molecular Medicine 16:2350–2361. 22304383 10.1111/j.1582-4934.2012.01545.x PMC3823428

[19] Martin F, Linden T, Katschinski DM, Oehme F, Flamme I, et al (2005) Copper-dependent activation of hypoxia-inducible factor (HIF)-1: implications for ceruloplasmin regulation. Blood 105:4613–4619. 15741220 10.1182/blood-2004-10-3980

[20] Okuyama H, Krishnamachary B, Zhou YF, Nagasawa H, Bosch-Marce M, et al (2006) Expression of vascular endothelial growth factor receptor 1 in bone marrow-derived mesenchymal cells is dependent on hypoxia-inducible factor 1. Journal of Biological Chemistry 281:15554–15563. 16574650 10.1074/jbc.M602003200

[21] Li S, Xie H, Li S, Kang YJ (2012) Copper stimulates growth of human umbilical vein endothelial cells in a vascular endothelial growth factor-independent pathway. Experimental Biology and Medicine 237:77–82. 22185917 10.1258/ebm.2011.011267

[22] Baldoli E, Maier JA (2012) Silencing TRPM7 mimics the effects of magnesium deficiency in human microvascular endothelial cells. Angiogenesis 15:47–57. 22183257 10.1007/s10456-011-9242-0

[23] Barralet J, Gbureck U, Habibovic P, Vorndran E, Gerard C, et al (2009) Angiogenesis in Calcium Phosphate Scaffolds by Inorganic Copper Ion Release. Tissue Engineering Part A 15:1601–1609. 19182977 10.1089/ten.tea.2007.0370

[24] Gérard C, Bordeleau L-J, Barralet J, Doillon CJ (2010) The stimulation of angiogenesis and collagen deposition by copper. Biomaterials 31:824–831. 19854506 10.1016/j.biomaterials.2009.10.009

[25] Frangoulis M, Georgiou P, Chrisostomidis C, Perrea D, Dontas I, et al (2007) Rat Epigastric Flap Survival and VEGF Expression after Local Copper Application. Plastic and reconstructive surgery 119:837–843. 17312485 10.1097/01.prs.0000252000.59231.5e

[26] Hidalgo-Bastida LA, Cartmell SH (2010) Mesenchymal stem cells, osteoblasts and extracellular matrix proteins: enhancing cell adhesion and differentiation for bone tissue engineering. Tissue Engineering Part B: Reviews 16:405–412. 20163206 10.1089/ten.TEB.2009.0714

[27] Hofmann A, Ritz U, Verrier S, Eglin D, Alini M, et al (2008) The effect of human osteoblasts on proliferation and neo-vessel formation of human umbilical vein endothelial cells in a long-term 3D co-culture on polyurethane scaffolds. Biomaterials 29:4217–4226. 18692894 10.1016/j.biomaterials.2008.07.024

[28] Kasten P, Beverungen M, Lorenz H, Wieland J, Fehr M, et al (2012) Comparison of Platelet-Rich Plasma and VEGF-Transfected Mesenchymal Stem Cells on Vascularization and Bone Formation in a Critical-Size Bone Defect. Cells Tissues Organs 196:523–533. 22796828 10.1159/000337490

[29] Kong N, Lin K, Li H, Chang J (2013) Synergy effects of copper and silicon ions on stimulation of vascularization by copper-doped calcium silicate. Journal of Materials Chemistry B: DOI: doi:10.1039/C1033TB21529F.. 32261627

[30] Marelli B, Ghezzi CE, Mohn D, Stark WJ, Barralet JE, et al (2011) Accelerated mineralization of dense collagen-nano bioactive glass hybrid gels increases scaffold stiffness and regulates osteoblastic function. Biomaterials 32:8915–8926. 21889796 10.1016/j.biomaterials.2011.08.016

[31] Yang L, Perez-Amodio S, Barrère-de Groot FYF, Everts V, van Blitterswijk CA, et al (2010) The effects of inorganic additives to calcium phosphate on in vitro behavior of osteoblasts and osteoclasts. Biomaterials 31:2976–2989. 20122718 10.1016/j.biomaterials.2010.01.002

[32] Wu C, Zhou Y, Fan W, Han P, Chang J, et al (2012) Hypoxia-mimicking mesoporous bioactive glass scaffolds with controllable cobalt ion release for bone tissue engineering. Biomaterials 33:2076–2085. 22177618 10.1016/j.biomaterials.2011.11.042

[33] Han P, Wu C, Xiao Y (2013) The effect of silicate ions on proliferation, osteogenic differentiation and cell signalling pathways (WNT and SHH) of bone marrow stromal cells. Biomaterials Science: DOI: doi:10.1039/C1032BM00108J. 32481903

[34] Contreras RG, Vilchis JRS, Sakagami H, Nakamura Y, Nakamura Y, et al (2010) Type of cell death induced by seven metals in cultured mouse osteoblastic cells. In Vivo 24:507–512. 20668317

[35] Fatokun AA, Stone TW, Smith RA (2008) Responses of differentiated MC3T3-E1 osteoblast-like cells to reactive oxygen species. European Journal of Pharmacology 587:35–41. 18448093 10.1016/j.ejphar.2008.03.024

[36] Kaigler D, Wang Z, Horger K, Mooney DJ, Krebsbach PH (2006) VEGF scaffolds enhance angiogenesis and bone regeneration in irradiated osseous defects. Journal of Bone and Mineral Research 21:735–744. 16734388 10.1359/jbmr.060120

[37] Santos MI, Reis RL (2010) Vascularization in bone tissue engineering: physiology, current strategies, major hurdles and future challenges. Macromolecular Bioscience 10:12–27. 19688722 10.1002/mabi.200900107

