# Supplementary figures and images for: Bioactive Copper-Doped Glass Scaffolds Can Stimulate Endothelial Cells in Co-Culture in Combination with Mesenchymal Stem Cells
Source: PLoS One. 2014 Dec 3;9(12):e113319. doi: 10.1371/journal.pone.0113319 (PMC4254617; doi:10.1371/journal.pone.0113319)

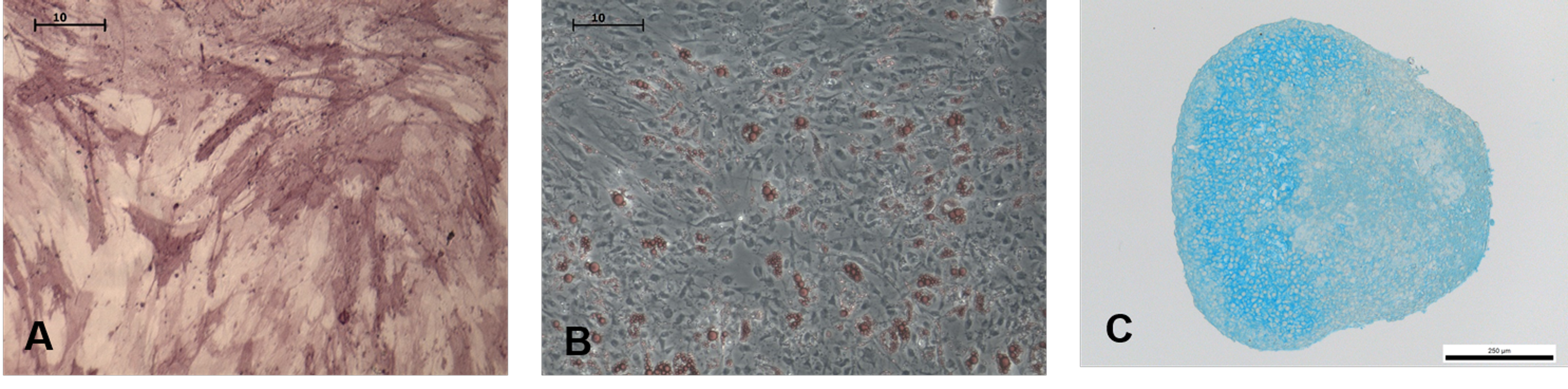

Supplement: Figure S1 — BMSCs are seeded in two-dimensional way in culture plastics. They are grown until 70% confluency and later differentiated into different lineages. After osteo-induction for 4 weeks, the cells are stained by alkaline phosphatase (A); after adipo-induction for 2 weeks, the cells are stained by oil red O (B); after chondrogenic induction for 2 weeks in a pellet culture, they are stained by alcian blue (C). (TIF) [file pone.0113319.s001.tif]

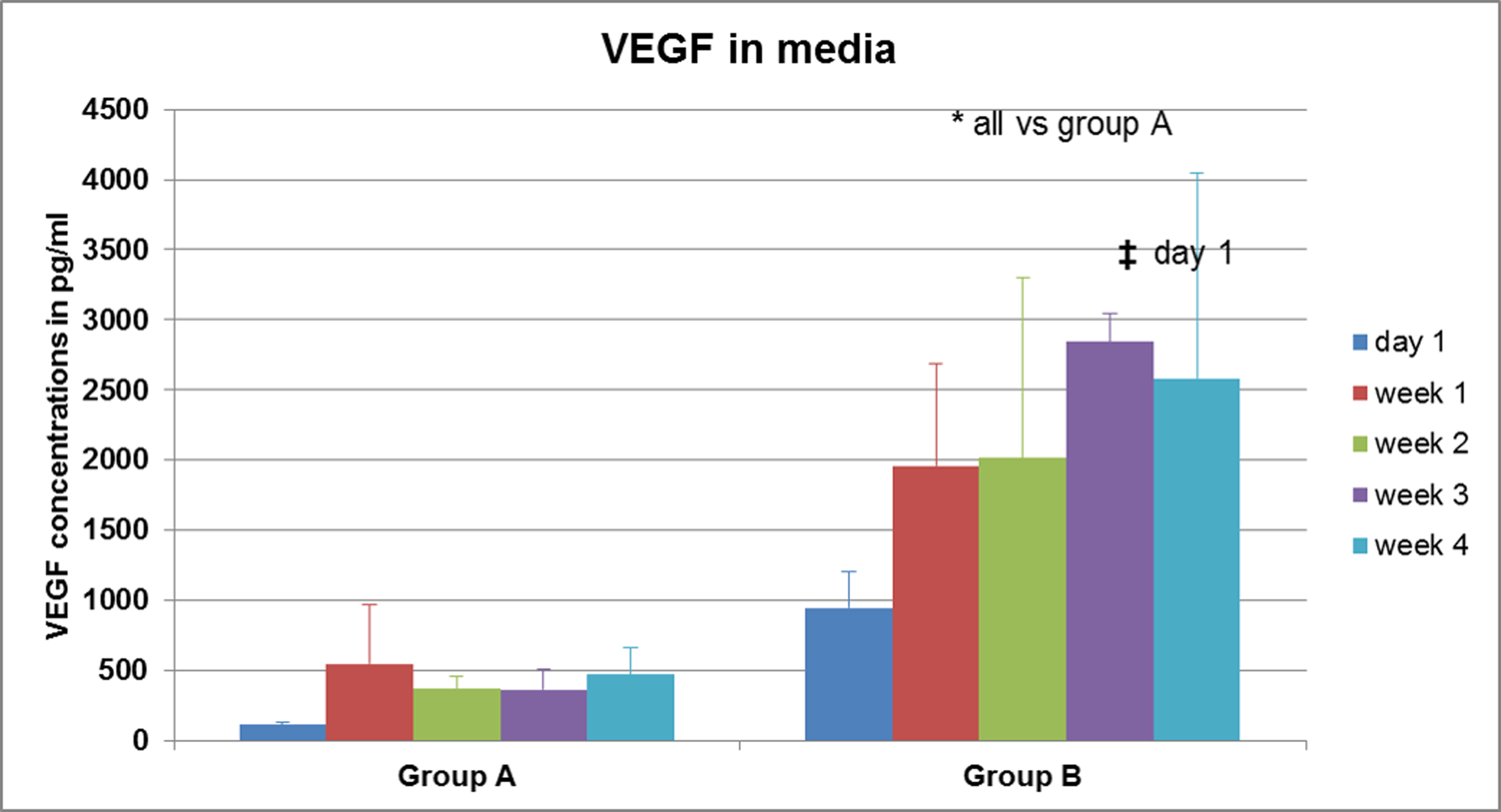

Supplement: Figure S2 — The supernatant media were collected from the MSC-BG (bio-active glass) constructs and were analyzed for VEGF concentration measured by ELISA. The significant results among different samples (time points) in same group is marked by ‡ and among same time point samples in different groups is marked by *. There was no adequate samples from Group C, for which the data for Group C are not presented. (TIF) [file pone.0113319.s002.tif]
